# 1317. Prevalence of *Pseudomonas aeruginosa *as the Causative Organism for Community Acquired Pneumonia

**DOI:** 10.1093/ofid/ofab466.1509

**Published:** 2021-12-04

**Authors:** Adam D Haviland, Wendy Szymczak, Gregory Weston

**Affiliations:** 1 Montefiore Medical Center, New York, New York; 2 Montefiore Medical Center, Albert Einstein College of Medicine, Bronx, NY; 3 Montefiore Medical Center and Albert Einstein College of Medicine, Bronx, New York

## Abstract

**Background:**

IDSA/ATS guidelines regarding pneumonia diagnosis and treatment changed in 2019. Guidelines recommend determining local prevalence of MRSA and *P. aeruginosa* to help guide empiric antibiotic coverage. The aim of our study was to determine the prevalence of *P. aeruginosa* as the causative organism for adult patients admitted to a large urban academic medical center with community acquired pneumonia (CAP).

**Methods:**

A report of urine streptococcus antigen tests collected January 1st-December 31st in 2019 was generated. Six hundred charts were reviewed and two hundred subjects met inclusion criteria (figure 1). Inclusion criteria were age >18, hospital admission, and documented suspicion of pneumonia by a physician.

**Results:**

The average age was 70 and half of the cases were women. The causative organism was identified in 60/200 cases (table 1). No cases of *P. aeruginosa* were identified. The most commonly isolated organisms were Influenza A and pneumococcus. 66% of cases had age >65yo, 25% were from long term care facilities, 34% had structural lung disease, 20% had dementia, 15% were hospitalized in the prior 90 days and received IV antibiotics, and 30% of cases met severe CAP criteria (table 2).

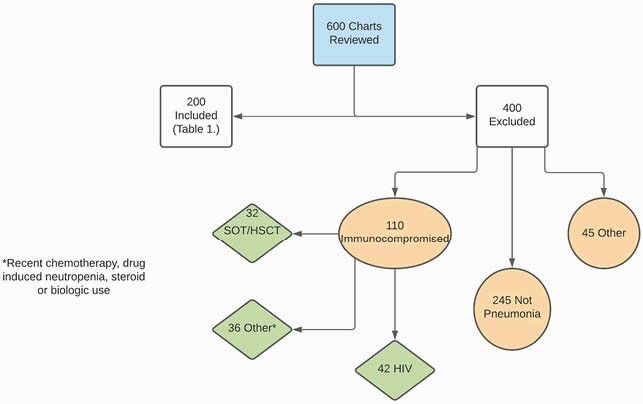

Figure 1. Workflow

Table 1. Organisms Identified

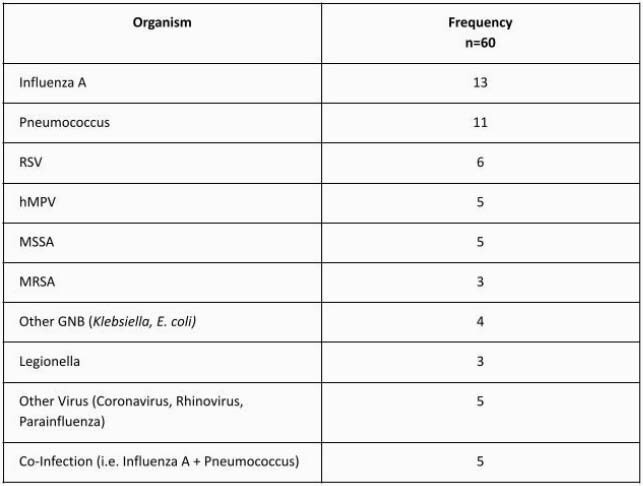

Table 2. Risk Factors

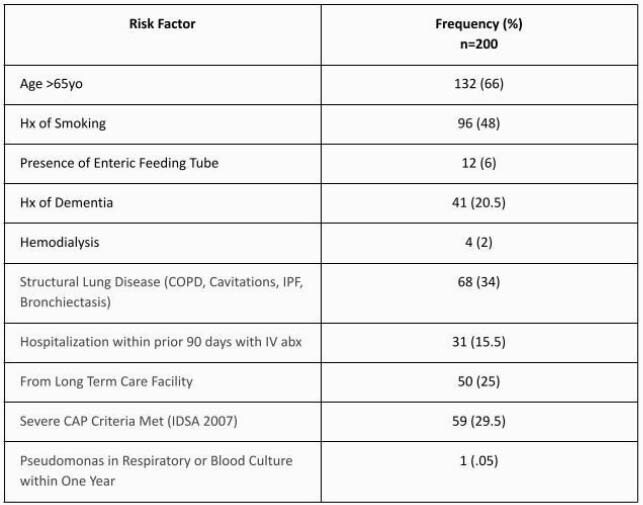

**Conclusion:**

Limitations include a low prevalence of renal failure in the study population, and lack of a standardized respiratory infection evaluation. Our results suggest that empiric coverage for *P. aeruginosa* may not be needed at our center in this cohort of older patients with clinical characteristics sometimes thought to be risk factors for *P. aeruginosa.*

**Disclosures:**

**Wendy Szymczak, PhD**, **Premier, Inc** (Consultant)**Qiagen** (Consultant, Scientific Research Study Investigator) **Gregory Weston, MD MSCR**, **Allergan** (Grant/Research Support)

